# Supplementary material for: The Mla pathway in Acinetobacter baumannii has no demonstrable role in anterograde lipid transport
Source: eLife. 2020 Sep 3;9:e56571. doi: 10.7554/eLife.56571 (PMC7500953; doi:10.7554/eLife.56571)
Supplement: Supplementary file 1. [file elife-56571-supp1.docx]

| **Locus** | **Fold-Change** | **FDR p-value** | **Annotation** |
| --- | --- | --- | --- |
| A1S_3103 | -6.74 | 0 | *mlaF* |
| A1S_2217 | -5.04 | 0 | *csuA* |
| A1S_2216 | -4.9 | 0 | *csuB* |
| A1S_2218 | -4.42 | 0 | *csuA/B* |
| A1S_2215 | -4.41 | 0 | *csuC* |
| A1S_2214 | -2.94 | 0 | *csuD* |
| A1S_0040 | -2.82 | 0 | Oxidoreductase |
| A1S_0041 | -2.57 | 0 | Linoleoyl-CoA Desaturase |
| A1S_2213 | -2.46 | 0 | *csuE* |
| A1S_2732 | -2.12 | 0 | Solanesyl diphosphate synthase |
| A1S_3755 | 2.08 | 2.29E-04 | Hypothetical protein |
| A1S_3613 | 2.35 | 0.02 | Hypothetical protein |
| A1S_3791 | 3.24 | 0 | Hypothetical protein |
| A1S_2889 | 4.4 | 0 | Hypothetical protein |

**Supplementary File 1: Differentially regulated genes between UGA Δ*mlaF* and WT**
