# Supplementary material for: The Mla pathway in Acinetobacter baumannii has no demonstrable role in anterograde lipid transport
Source: eLife. 2020 Sep 3;9:e56571. doi: 10.7554/eLife.56571 (PMC7500953; doi:10.7554/eLife.56571)
Supplement: Supplementary file 2. [file elife-56571-supp2.docx]

**Supplementary File 2: Differentially regulated genes between UW Δ*mlaF* and WT**

| **Locus** | **Fold-Change** | **FDR p-value** | **Annotation** |
| --- | --- | --- | --- |
| A1S_3103 | -7.9 | 0 | *mlaF* |
| A1S_1147 | -6.21 | 0.02 | Hypothetical protein |
| A1S_3102 | -4.63 | 0 | *mlaE* |
| A1S_0067 | -3.94 | 0 | L-Lactate Permease |
| A1S_3568 | -3.91 | 0 | Hypothetical protein |
| A1S_0371 | -3.8 | 0 | Hypothetical protein |
| A1S_0683 | -3.73 | 0 | *raiA* |
| A1S_1932 | -3.68 | 0 | Hypothetical protein |
| A1S_3564 | -3.62 | 0 | Hypothetical protein |
| A1S_0070 | -3.49 | 0 | D-lactate dehydrogenase |
| A1S_3580 | -3.39 | 0 | Hypothetical protein |
| A1S_2230 | -3.33 | 0 | Hypothetical protein |
| A1S_0069 | -3.26 | 0 | L-lactate dehydrogenase |
| A1S_0800 | -3.19 | 0 | Bacterioferritin |
| A1S_3797 | -3.07 | 0 | Hypothetical protein |
| A1S_3175 | -3.05 | 0 | Bacterioferritin |
| A1S_1950 | -2.94 | 0 | Hypothetical protein |
| A1S_2809 | -2.88 | 0 | *ecnB* |
| A1S_3641 | -2.87 | 0 | Hypothetical protein |
| A1S_3661 | -2.79 | 0 | Hypothetical protein |
| A1S_1224 | -2.7 | 0 | Transposase |
| A1S_0068 | -2.67 | 0 | L-Lactate Utilization Repressor |
| A1S_2183 | -2.62 | 0 | Hypothetical protein |
| A1S_2093 | -2.62 | 0 | Hypothetical protein |
| A1S_3658 | -2.55 | 1.97E-12 | Hypothetical protein |
| A1S_3099 | -2.54 | 0 | *mlaB* |
| A1S_3750 | -2.53 | 0 | Hypothetical protein |
| A1S_1680 | -2.51 | 0 | Hypothetical protein |
| A1S_3350 | -2.5 | 0 | Hypothetical protein |
| A1S_3024 | -2.48 | 0 | Hypothetical protein |
| A1S_0445 | -2.47 | 0 | Hypothetical protein |
| A1S_1687 | -2.45 | 0 | Hypothetical protein |
| A1S_2654 | -2.44 | 0 | Hypothetical protein |
| A1S_3864 | -2.4 | 0 | Hypothetical protein |
| A1S_3629 | -2.39 | 0 | Hypothetical protein |
| A1S_3101 | -2.35 | 0 | *mlaC* |
| A1S_3679 | -2.35 | 0 | Hypothetical protein |
| A1S_3146 | -2.3 | 0 | Hypothetical protein |
| A1S_2330 | -2.27 | 0 | Hypothetical protein |
| A1S_1925 | -2.27 | 0 | *cydB* |
| A1S_0771 | -2.25 | 0 | Hypothetical protein |
| A1S_1926 | -2.25 | 0 | Hypothetical protein |
| A1S_2414 | -2.24 | 0 | Hypothetical protein |
| A1S_2331 | -2.22 | 0 | *fadE* |
| A1S_2961 | -2.18 | 0 | Hypothetical protein |
| A1S_0779 | -2.16 | 0 | Hypothetical protein |
| A1S_2939 | -2.09 | 0 | *actP* |
| A1S_1433 | -2.06 | 0 | *cydB* |
| A1S_1522 | -2.06 | 0 | Hypothetical protein |
| A1S_3298 | -2.05 | 0 | Hypothetical protein |
| A1S_0372 | -2.04 | 0 | Hypothetical protein |
| A1S_1042 | -2.04 | 0 | Hypothetical protein |
| A1S_3914 | -2.03 | 0 | Hypothetical protein |
| A1S_3532 | 2.0 | 0 | Hypothetical protein |
| A1S_1466 | 2.03 | 0 | *aspQ* |
| A1S_3553 | 2.05 | 0 | Hypothetical protein |
| A1S_0087 | 2.08 | 0 | Hypothetical protein |
| A1S_1366 | 2.12 | 0 | Hypothetical protein |
| A1S_3719 | 2.18 | 0 | Hypothetical protein |
| A1S_0641 | 2.2 | 0 | Hypothetical protein |
| A1S_3545 | 2.21 | 0 | Hypothetical protein |
| A1S_3522 | 2.23 | 0 | Hypothetical protein |
| A1S_3402 | 2.24 | 0 | Hypothetical protein |
| A1S_3273 | 2.26 | 0 | Hypothetical protein |
| A1S_3657 | 2.26 | 1.79E-9 | Hypothetical protein |
| A1S_0650 | 2.29 | 0 | Hypothetical protein |
| A1S_3523 | 2.4 | 0 | Hypothetical protein |
| A1S_0630 | 2.42 | 0 | Hypothetical protein |
| A1S_3691 | 2.42 | 0.01 | Hypothetical protein |
| A1S_0633 | 2.45 | 0 | Hypothetical protein |
| A1S_0631 | 2.51 | 0 | Hypothetical protein |
| A1S_3174 | 2.54 | 0 | Hypothetical protein |
| A1S_3544 | 2.55 | 0 | Hypothetical protein |
| A1S_3656 | 2.59 | 0 | Hypothetical protein |
| A1S_0643 | 2.62 | 0 | Hypothetical protein |
| A1S_3534 | 2.68 | 0 | Hypothetical protein |
| A1S_0526 | 2.7 | 0 | Hypothetical protein |
| A1S_0640 | 2.71 | 0 | Hypothetical protein |
| A1S_0647 | 2.73 | 0 | *icmO* |
| A1S_0646 | 2.8 | 0 | *icmB* |
| A1S_2162 | 2.86 | 0 | *ycdS* |
| A1S_3908 | 2.9 | 0 | Hypothetical protein |
| A1S_0642 | 2.97 | 0 | Hypothetical protein |
| A1S_3543 | 3.02 | 0 | Hypothetical protein |
| A1S_3512 | 3.04 | 0.01 | Hypothetical protein |
| A1S_3542 | 3.09 | 0 | Hypothetical protein |
| A1S_3591 | 3.34 | 0 | Hypothetical protein |
| A1S_3541 | 3.41 | 0 | Hypothetical protein |
| A1S_3546 | 3.44 | 0 | Hypothetical protein |
| A1S_0644 | 3.5 | 0 | Hypothetical protein |
| A1S_3552 | 3.69 | 0 | Hypothetical protein |
| A1S_3907 | 3.73 | 2.02E-4 | Hypothetical protein |
| A1S_3539 | 3.76 | 0 | Hypothetical protein |
| A1S_3540 | 3.8 | 0 | Hypothetical protein |
| A1S_3535 | 3.83 | 0 | Hypothetical protein |
| A1S_0645 | 3.9 | 0 | Hypothetical protein |
| A1S_2213 | 6.17 | 0 | *csuE* |
| A1S_2214 | 6.49 | 0 | *csuD* |
| A1S_3796 | 7.17 | 0 | Hypothetical protein |
| A1S_2215 | 7.39 | 0 | *csuC* |
| A1S_2217 | 8.3 | 0 | *csuA* |
| A1S_2216 | 8.63 | 0 | *csuB* |
| A1S_2218 | 8.94 | 0 | *csuA/B* |
