# Supplementary material for: The Mla pathway in Acinetobacter baumannii has no demonstrable role in anterograde lipid transport
Source: eLife. 2020 Sep 3;9:e56571. doi: 10.7554/eLife.56571 (PMC7500953; doi:10.7554/eLife.56571)
Supplement: Supplementary file 3. [file elife-56571-supp3.docx]

**Supplementary File 3: Unique mutations present in Δ*mlaC, obgE::kan,* pMMB67EH-*obgE****

| **Gene** | **Mutation** | **Impact** | **Frequency** | **Function** |
| --- | --- | --- | --- | --- |
| A1S_0238 | C>T | Q137S | 100% | *thrC* |
| A1S_1979 | Insertion >T | *K84fs | 100% | *adeN* |
|  | A > Deletion | *K15fs | 100% | *adeN* |
| A1S_2858 | T > Deletion | N104fs | 100% | Hypothetical methyltransferase |

*****Identified in one of two mutants generated
