# Supplementary material for: The Mla pathway in Acinetobacter baumannii has no demonstrable role in anterograde lipid transport
Source: eLife. 2020 Sep 3;9:e56571. doi: 10.7554/eLife.56571 (PMC7500953; doi:10.7554/eLife.56571)
Supplement: Supplementary file 4. [file elife-56571-supp4.docx]

**Supplementary File 4: Strains and plasmids used in this study**

| **Strain** | **Genotype** | **Reference** |
| --- | --- | --- |
| *A. baumannii* 17978 (UGA) | Wild Type | ATCC |
| *A. baumannii* 17978 (UW) | Wild Type | 33 |
| *A. baumannii* 17978 (UGA) | Δ*mlaF* | This study |
| *A. baumannii* 17978 (UW) | Δ*mlaF* | 33 |
| *A. baumannii* 17978 (UW) | *mlaC::kan* | This study |
| *A. baumannii* 17978 (UW) | *mlaF::kan* | This study |
| *E. coli* W3110 | WT, F- λ-, *rph-1 IN*(*rrnD,rrnE*)1 | *E. coli* Genetic Stock Center (Yale) |
| *A. baumannii* 17978 (UW) | *obgE::kan*, pMMB67EH-*obgE** | This study |
| *A. baumannii* 17978 (UW) | Δ*mlaC, obgE::kan,* pMMB67EH-*obgE** and mutations described in Table S3 | This study |
| **Plasmid** | **Description** | **Reference** |
| pMMB67EH-Kn | pMMB67EH with Kan^R^ | 46 |
| pMMB67EH-Hyg | pMMB67EH with Hyg^R^ | This study |
| pRecAB | pMMB67EH with REC_AB_ system | 46 |
| pFLP | pMMB67EH with FLP recombinase | 46 |
| pKD4 | FRT-flanked Kan^R^ | 46 |
| pMMB67EH-*obgE** | pMMB67EH-Hyg with *obgE** | This study |
