# Supplementary material for: The Mla pathway in Acinetobacter baumannii has no demonstrable role in anterograde lipid transport
Source: eLife. 2020 Sep 3;9:e56571. doi: 10.7554/eLife.56571 (PMC7500953; doi:10.7554/eLife.56571)
Supplement: Supplementary file 5. [file elife-56571-supp5.docx]

**Supplementary File 5: Primers used in this study**

| **Primer** | **Sequence** |
| --- | --- |
| *mlaC::kan*  *recombineering_F* | 5’-GGTGGTGGCGCAGGTAAGGTTGCCGCTGGTTCATCCTCAGCAGAAGAAAAAGCTCCTGCAAGTACTGACAGCAGTGCACAGCCGTCATTTGTTGAGTAAGAGATTATTTGAAGGAGTGGTTCCAGAGCGATTGTGTAGGCTGGAGCTGCTTCG |
| *mlaC::kan*  *recombineering_R* | 5’- CAATTTTGCCTGAAACAACTAACTCTTGGTTAAGATACTGAACCACTCTTCACCTACTTATTTTTGTTTATTCTGATTAACAGCAGCATCTGCATCAGGTTGGAACGTAGCAATTGCTTTATTCAATATCCTCCTTAGTTCCTATTCCG |
| *mlaF::kan*  *recombineering_F* | 5’-TCGATCAATAAATTTCTTTTCAAAATCTAGCTCATATTCCTATATTAGCTTGTTTTGCTTTTGTCAAAATTACAACAAGATTCCCAAAATTCAGTGTTATAGTATGCGGGCATAAAAGTGCAGGAAGCGATTGTGTAGGCTGGAGCTGCTTCG |
| *mlaF::kan*  *recombineering_R* | 5’- GACGAAACGTCCAAAACCGCCCGCTGAAGGTAATGAAAAAATAATCTGTAAGAGCATCAGTGCCGCAACACCAATCCCTCGAATCCGCTCAATAACGAGTCTACCTAACCAGGCAATCGTATTCATATCCTCCTTAGTTCCTATTCCG |
| *obgE::kan*  *recombineering_F* | 5’-TTTATATCCTGATAAAAAGAAATAAAATAAGCAGCAGGAAAGTAGAATTCTCTCGATTTTCTATTCGAGAATTTTCTGAAAATATGGCAGAATAGGCGGTTTTAGAGTTTTTAGAGGATTGGCAAAGCGATTGTGTAGGCTGGAGCTGCTTCG |
| *obgE::kan*  *recombineering_R* | 5’ -TCCAGATCTAGACCCTGCCCATTTGCCGTGAGTAAAGATGATCCGATTTTAACAACGATTCGTTTACACTCACTGAGCTTACGTTGCCCATCGACCACTTCTATCATGTTTTCCTCGGTTTTTTATATCCTCCTTAGTTCCTATTCCG |
| *obgE-pMMB_F* | 5’ -CCG**GAATTC**ATGCGCTTTGTTGATGAAGC |
| *obgE-pMMB_R* | 5’ - CGC**GGATCC**TTAATCACGAACGTAGATGCTTTC |
